# Supplementary material for: Using affective knowledge to generate and validate a set of emotion-related, action words
Source: PeerJ. 2015 Jul 28;3:e1100. doi: 10.7717/peerj.1100 (PMC4525695; doi:10.7717/peerj.1100)
Supplement: Appendix S1 — Alphabetised, full set of modal action words, generated by two of more participants during study 1 (N = 25). Unmerged response frequencies, per emotion label, are shown. Responses regarded as synonymous, via our criteria, are labelled as ‘core’ or ‘subsidiary’ and corresponding core or subsidiary action words are provided in the final column. [file peerj-03-1100-s001.docx]

*Appendix A.*

*Table 6: Alphabetised, full set of modal action words, generated by two of more participants during study 1 (N=25). Unmerged response frequencies, per emotion label, are shown. Responses regarded as synonymous, via our criteria, are labelled as ‘core’ or ‘subsidiary’ and corresponding core or subsidiary action words are provided in the final column.*

|  |  | ***Sad*** | ***Happy*** | ***Anger*** | ***Disgust*** | ***Fear*** | ***Surprise*** | ***Core/Subsidiary*** | ***Synonymous core/subsidiary exemplar(s)*** |
| --- | --- | --- | --- | --- | --- | --- | --- | --- | --- |
| *Attack* |  | *0* | *0* | *2* | *0* | *0* | *0* |  |  |
| *Avoid* |  | *0* | *0* | *0* | *4* | *2* | *0* | *Subsidiary* | *Hide* |
| *Beat* |  | *0* | *0* | *2* | *0* | *0* | *0* |  |  |
| *Break* |  | *0* | *0* | *2* | *0* | *0* | *0* |  |  |
| *Cheer* |  | *0* | *3* | *0* | *0* | *0* | *0* | *Subsidiary* | *Clap* |
| *Clap* |  | *0* | *4* | *0* | *0* | *0* | *0* | *Core* | *Cheer* |
| *Clench* |  | *0* | *0* | *3* | *0* | *0* | *0* |  |  |
| *Cower* |  | *0* | *0* | *0* | *0* | *2* | *0* |  |  |
| *Cringe* |  | *0* | *0* | *0* | *2* | *0* | *0* | *Subsidiary* | *Recoil* |
| *Cry* |  | *18* | *6* | *7* | *0* | *9* | *3* | *Core* | *Sob; Weep* |
| *Dance* |  | *0* | *6* | *0* | *0* | *0* | *0* | *Core* | *Skip* |
| *Exclaim* |  | *0* | *0* | *0* | *0* | *0* | *2* |  |  |
| *Fight* |  | *0* | *0* | *4* | *0* | *0* | *0* |  |  |
| *Frown* |  | *9* | *0* | *3* | *4* | *0* | *0* | *Core* | *Grimace* |
| *Gag* |  | *0* | *0* | *0* | *3* | *0* | *0* | *Core* | *Retch* |
| *Gape (originally ‘open mouth’)* |  | *0* | *0* | *0* | *0* | *0* | *2* |  |  |
| *Gasp* |  | *0* | *0* | *0* | *0* | *2* | *8* | *Core* | *Inhale; Sharp Intake* |
|  |  | ***Sad*** | ***Happy*** | ***Anger*** | ***Disgust*** | ***Fear*** | ***Surprise*** | ***Core/Subsidiary*** | ***Synonymous Core/Subsidiary Exemplar(s)*** |
| *Giggle* |  | *0* | *2* | *0* | *0* | *0* | *0* | *Subsidiary* | *Laugh* |
| *Grimace* |  | *0* | *0* | *0* | *2* | *0* | *0* | *Subsidiary* | *Frown* |
| *Grin* |  | *0* | *6* | *0* | *0* | *0* | *2* | *Subsidiary* | *Smile* |
| *Growl* |  | *0* | *0* | *4* | *0* | *0* | *0* |  |  |
| *Hide* |  | *2* | *0* | *0* | *0* | *11* | *0* | *Core* | *Avoid* |
| *Hit* |  | *0* | *0* | *9* | *0* | *0* | *0* | *Core* | *Punch* |
| *Hug* |  | *0* | *8* | *0* | *0* | *0* | *0* |  |  |
| *Hum* |  | *0* | *2* | *0* | *0* | *0* | *0* |  |  |
| *Hurt* |  | *2* | *0* | *0* | *0* | *0* | *0* |  |  |
| *Ignore* |  | *0* | *0* | *0* | *2* | *0* | *0* |  |  |
| *Inhale* |  | *0* | *0* | *0* | *0* | *0* | *2* | *Subsidiary* | *Gasp* |
| *Isolate* |  | *2* | *0* | *0* | *0* | *0* | *0* |  |  |
| *Joke* |  | *0* | *2* | *0* | *0* | *0* | *0* | *Core* | *Play* |
| *Jump* |  | *0* | *2* | *0* | *0* | *5* | *15* |  |  |
| *Kick* |  | *0* | *0* | *5* | *0* | *0* | *0* |  |  |
| *Laugh* |  | *0* | *20* | *0* | *0* | *0* | *11* | *Core* | *Giggle* |
| *Mope* |  | *2* | *0* | *0* | *0* | *0* | *0* |  |  |
| *Panic* |  | *0* | *0* | *0* | *0* | *7* | *0* |  |  |
| *Play* |  | *0* | *2* | *0* | *0* | *0* | *0* | *Subsidiary* | *Joke* |
| *Punch* |  | *0* | *0* | *4* | *0* | *0* | *0* | *Subsidiary* | *Hit* |
| *Rage* |  | *0* | *0* | *2* | *0* | *0* | *0* | *Subsidiary* | *Rant* |
| *Raise Eyebrow* |  | *0* | *0* | *0* | *0* | *0* | *3* |  |  |
| *Rant* |  | *0* | *0* | *4* | *0* | *0* | *0* | *Core* | *Rage* |
| *Recoil* |  | *0* | *0* | *0* | *5* | *0* | *0* | *Core* | *Cringe* |
|  |  | ***Sad*** | ***Happy*** | ***Anger*** | ***Disgust*** | ***Fear*** | ***Surprise*** | ***Core/Subsidiary*** | ***Synonymous Core/Subsidiary Exemplar(s)*** |
| *Retch* |  | *0* | *0* | *0* | *2* | *0* | *0* | *Subsidiary* | *Gag* |
| *Run* |  | *0* | *0* | *0* | *0* | *13* | *0* |  |  |
| *Scream* |  | *0* | *0* | *17* | *0* | *7* | *4* | *Core* | *Shout; Shriek, Yell* |
| *Shake* |  | *0* | *0* | *6* | *0* | *8* | *0* | *Core* | *Shiver* |
| *Sharp intake* |  | *0* | *0* | *0* | *0* | *0* | *3* | *Subsidiary* | *Inhale* |
| *Shiver* |  | *0* | *0* | *0* | *0* | *3* | *0* | *Subsidiary* | *Shake* |
| *Shout* |  | *0* | *0* | *13* | *0* | *0* | *4* | *Subsidiary* | *Scream* |
| *Shriek* |  | *0* | *0* | *0* | *0* | *0* | *2* | *Subsidiary* | *Scream* |
| *Shudder* |  | *0* | *0* | *0* | *2* | *0* | *0* |  |  |
| *Sigh* |  | *2* | *0* | *0* | *0* | *0* | *0* |  |  |
| *Sing* |  | *0* | *5* | *0* | *0* | *0* | *0* |  |  |
| *Skip* |  | *0* | *4* | *0* | *0* | *0* | *0* | *Subsidiary* | *Dance* |
| *Sleep* |  | *3* | *0* | *0* | *0* | *0* | *0* |  |  |
| *Slow* |  | *2* | *0* | *0* | *0* | *0* | *0* |  |  |
| *Smile* |  | *0* | *21* | *0* | *0* | *0* | *4* | *Core* | *Grin* |
| *Sneer* |  | *0* | *0* | *0* | *3* | *0* | *0* |  |  |
| *Sob* |  | *3* | *0* | *0* | *0* | *0* | *0* |  |  |
| *Socialise* |  | *0* | *2* | *0* | *0* | *0* | *0* |  |  |
| *Squeal* |  | *0* | *0* | *0* | *0* | *0* | *2* |  |  |
| *Stomp* |  | *0* | *0* | *2* | *0* | *0* | *0* |  |  |
| *Stutter* |  | *0* | *0* | *0* | *0* | *2* | *0* |  |  |
| *Swear* |  | *0* | *0* | *3* | *0* | *0* | *0* |  |  |
| *Sweat* |  | *0* | *0* | *0* | *0* | *4* | *0* |  |  |
|  |  | ***Sad*** | ***Happy*** | ***Anger*** | ***Disgust*** | ***Fear*** | ***Surprise*** | ***Core/Subsidiary*** | ***Synonymous Core/Subsidiary Exemplar(s)*** |
| *Tense* |  | *0* | *0* | *2* | *0* | *4* | *0* |  |  |
| *Throw* |  | *0* | *0* | *4* | *0* | *0* | *0* |  |  |
| *Vomit* |  | *0* | *0* | *0* | *5* | *0* | *0* |  |  |
| *Wallow* |  | *2* | *0* | *0* | *0* | *0* | *0* |  |  |
| *Weep* |  | *2* | *0* | *0* | *0* | *0* | *0* |  |  |
| *Withdraw* |  | *7* | *0* | *0* | *4* | *2* | *0* |  |  |
| *Yell* |  | *0* | *0* | *4* | *0* | *0* | *2* | *Subsidiary* | *Scream* |
| *Yelp* |  | *0* | *0* | *0* | *0* | *0* | *2* |  |  |
