# Supplementary material for: Using affective knowledge to generate and validate a set of emotion-related, action words
Source: PeerJ. 2015 Jul 28;3:e1100. doi: 10.7717/peerj.1100 (PMC4525695; doi:10.7717/peerj.1100)
Supplement: Appendix S2 — Note that ‘C-to-A’ represents the category-to-action word rating direction, and ‘A-to-C’ the action word-to-category rating direction. Modal pairings are presented in bold. [file peerj-03-1100-s002.docx]

*Appendix B:*

*Table 7: Mean ratings (SD) for action word and label pairings, by direction rating and instruction perspective. Note that ‘C-to-A’ represents the category-to-action word rating direction, and ‘A-to-C’ the action word-to-category rating direction. Modal pairings are presented in bold.*

|  |  | **Scream** | | **Smile** | | **Jump** | | **Cry** | | | **Recoil** | | **Hide** | | | **Totals** |
| --- | --- | --- | --- | --- | --- | --- | --- | --- | --- | --- | --- | --- | --- | --- | --- | --- |
|  |  | C-to-A | A-to-C | C-to-A | A-to-C | C-to-A | A-to-C | C-to-A | A-to-C | | C-to-A | A-to-C | C-to-A | A-to-C | |  |
| **First Person Perspective** | **Anger** | **3.79 (1.18)** | **3.90 (1.21)** | 1.37 (0.60) | 2.00 (0.79) | 1.79 (0.98) | 2.10 (1.12) | 3.11 (1.45) | 3.35 (1.46) | | 2.11 (1.10) | 2.75 (1.29) | 2.47 (1.31) | 2.90 (1.21) | | 2.64 (1.41) |
|  | **Happy** | 2.32 (1.20) | 2.75 (1.16) | **4.68 (0.67)** | **4.75 (0.44)** | 2.63 (1.37) | 3.10 (1.17) | 2.26 (1.33) | 2.15 (1.18) | | 1.42 (0.77) | 1.45 (0.60) | 1.53 (1.07) | 1.75 (0.85) | | 2.57 (0.98) |
|  | **Surprise** | 3.53 (1.31) | 3.75 (1.12) | 2.89 (1.27) | 3.60 (0.99) | **3.95 (1.27)** | **4.10 (1.12)** | 2.42 (1.17) | 2.30 (1.22) | | 2.79 (1.13) | 3.85 (0.88) | 2.12 (1.10) | 1.80 (0.83) | | 3.09 (1.12) |
|  | **Sad** | 1.63 (0.68) | 2.00 (1.12) | 1.42 (0.69) | 2.05 (1.00) | 1.26 (0.45) | 1.45 (0.69) | **4.00 (1.33)** | **4.75 (0.44)** | | 2.26 (1.19) | 2.05 (1.00) | 3.37 (1.46) | 3.50 (1.43) | | 2.48 (0.96) |
|  | **Disgust** | 3.11 (1.24) | 2.30 (1.34) | 1.53 (0.90) | 1.25 (0.55) | 2.58 (1.17) | 1.75 (1.02) | 2.37 (1.30) | 2.40 (1.23) | | **3.58 (1.22)** | **4.45 (0.77)** | 2.37 (1.07) | 2.00 (1.07) | | 2.47 (1.07) |
|  | **Fear** | 3.63 (1.21) | 3.60 (1.23) | 1.63 (0.68) | 1.45 (0.69) | 3.21 (1.55) | 3.05 (1.61) | 3.16 (1.34) | 2.90 (1.37) | | 3.47 (1.12) | 3.95 (0.94) | **3.79 (1.23)** | **4.40 (0.50)** | | 3.19 (1.12) |
|  | **Totals** | 3.00 (1.14) | 3.05 (1.20) | 2.51 (0.80) | 2.52 (0.74) | 2.57 (0.13) | 2.59 (1.12) | 2.89 (1.32) | 2.98 (1.15) | | 2.61 (1.26) | 3.08 (0.91) | 2.61 (1.21) | 2.73 (0.98) | | - |
| **Third Person Perspective** | **Anger** | **4.00 (0.73)** | **4.15 (0.93)** | 1.60 (0.88) | 1.50 (0.61) | 1.90 (0.85) | 2.50 (1.15) | 3.20 (0.89) | 3.05 (1.23) | | 1.95 (0.83) | 2.95 (1.10) | 2.15 (1.04) | 2.65 (0.99) | | 2.63 (0.94) |
|  | **Happy** | 2.65 (1.35) | 3.55 (1.10) | **4.85 (0.37)** | **4.84 (0.37)** | 3.00 (1.30) | 3.40 (1.23) | 2.75 (1.33) | 3.20 (1.01) | | 1.30 (0.57) | 1.55 (0.83) | 1.30 (0.47) | 1.80 (0.83) | | 2.85 (0.90) |
|  | **Surprise** | 3.65 (0.81) | 3.95 (0.76) | 3.30 (1.08) | 3.30 (1.08) | **4.00 (1.12)** | **4.25 (0.97)** | 3.00 (0.92) | 2.75 (0.97) | | 3.45 (0.88) | 3.40 (1.43) | 2.55 (1.23) | 2.05 (0.76) | | 3.31 (1.00) |
|  | **Sad** | 2.55 (1.19) | 2.15 (0.99) | 1.40 (0.50) | 1.55 (0.76) | 1.45 (0.60) | 1.80 (0.95) | **4.40 (0.50)** | **4.70 (0.47)** | | 2.60 (1.19) | 1.65 (0.93) | 3.80 (0.83) | 3.80 (0.77) | | 2.65 (0.81) |
|  | **Disgust** | 2.80 (0.95) | 3.15 (1.23) | 1.20 (0.41) | 1.45 (0.76) | 2.15 (0.99) | 2.50 (1.19) | 2.30 (0.86) | 2.70 (1.13) | | **4.40 (0.60)** | **4.35 (1.23)** | 2.60 (1.10) | 2.45 (1.10) | | 2.67 (0.96) |
|  | **Fear** | 3.95 (0.60) | 4.15 (0.81) | 1.45 (0.51) | 1.60 (0.75) | 3.45 (1.15) | 3.75 (1.29) | 4.00 (0.73) | 3.80 (0.77) | | 3.90 (0.85) | 4.20 (1.06) | **4.45 (0.51)** | **4.75 (0.44)** | | 3.62 (0.79) |
|  | **Totals** | 2.93  (0.94) | 3.52 (0.97) | 2.30 (0.63) | 2.54 (0.72) | 2.66 (1.00) | 3.03 (1.13) | 3.28 (0.87) | 3.37 (0.93) | 2.93 (0.82) | | 3.02 (1.10) | 2.81 (0.86) | 2.92 (0.82) | - | |
